# Exploring the Potential of *Haematococcus pluvialis* as a Source of Bioactives for Food Applications: A Review

**DOI:** 10.3390/microorganisms13112606

**Published:** 2025-11-16

**Authors:** Joseane C. Bassani, Sthéfani da Cunha, Deborah Catharine de Assis Leite, Creciana M. Endres, Crivian Pelisser, Karine L. Meneghetti, Gabriel Bombo, Alcina M. M. B. Morais, Rui M. S. C. Morais, Geciane T. Backes, Juliana Steffens

**Affiliations:** 1 Department of Agricultural Sciences, Regional Integrated University of Alto Uruguai and the Missions (URI)—Erechim Campus, Erechim 99709-910, RS, Brazil; joseanebassani@gmail.com (J.C.B.); gtoniazzo@uricer.edu.br (G.T.B.); julisteffens@uricer.edu.br (J.S.); 2 SENAI Institute of Technology for Food and Beverages, Chapecó 89803-000, SC, Brazil; sthefanicunhaa@gmail.com (S.d.C.); karine.meneghetti@sc.senai.br (K.L.M.); 3 Federal Technological University of Paraná—Dois Vizinhos Campus, Dois Vizinhos 85660, PR, Brazil; deborahleite@utfpr.edu.br; 4 Centro Universitário SENAI Santa Catarina—UniSENAI, Campus Chapecó, Chapecó 89803-800, SC, Brazil; crivian.pelisser@edu.sc.senai.br; 5 GreenCoLab, University of Algarve, 8005-139 Faro, Portugal; gabrielbombo@greencolab.com; 6 CBQF—Centro de Biotecnologia e Química Fina—Laboratório Associado, Escola Superior de Biotecnologia, Universidade Católica Portuguesa, Rua Diogo Botelho 1327, 4169-005 Porto, Portugal; abmorais@ucp.pt (A.M.M.B.M.); rcmorais@ucp.pt (R.M.S.C.M.)

**Keywords:** *Haematococcus pluvialis*, astaxanthin, microalgae, bioactives, functional foods

## Abstract

The search for sustainable and health-promoting food ingredients has positioned microalgae as promising candidates for the development of functional products. *Haematococcus pluvialis*, a unicellular green microalga, is the richest natural source of astaxanthin, a carotenoid with outstanding antioxidant, anti-inflammatory, and neuroprotective properties. In addition to astaxanthin, *H. pluvialis* provides high-value proteins, essential fatty acids, polysaccharides, and vitamins, which expand its potential applications in the food sector. This review compiles current knowledge on the biology and physiology of *H. pluvialis*, with emphasis on cultivation strategies, environmental stress factors, and biotechnological tools designed to enhance bioactive compound production. Advances in extraction and purification methods are also discussed, contrasting conventional solvent-based approaches with emerging green technologies. The integration of these strategies with biomass valorization highlights opportunities for improving economic feasibility and sustainability. Applications of *H. pluvialis* in the food industry include its use as a functional ingredient, natural colorant, antioxidant, and stabilizer in bakery products, beverages, meat analogs, and emulsified systems. Evidence from in vitro, in vivo, and clinical studies reinforces its safety and effectiveness. Looking ahead, industrial perspectives point to the adoption of omics-based tools, metabolic engineering, and circular economy approaches as drivers to overcome current barriers of cost, stability, and regulation, opening new avenues for large-scale applications in food systems.

## 1. Introduction

One of the major challenges to be faced in the coming decades is ensuring food production and safety for a population that is growing at an alarmingly fast pace. According to data from the United Nations, the global population increases by approximately 83 million people annually. It is estimated that by 2030, the world population will reach between 8.4 and 8.6 billion inhabitants, and by 2050, it will range between 9.4 and 10.2 billion [1]. This number will result in an 88% increase in demand for protein to feed the population, which will put the planet’s biocapacity at risk. Therefore, concern for the preservation of natural resources must be aligned with the need to feed an ever-growing population, and the use of microalgae in food emerges as a highly promising approach [2].

Traditional protein sources, such as animal products and conventional agricultural crops, face significant environmental and economic limitations. Among the main challenges are soil degradation, high water consumption, and significant greenhouse gas emissions. The scarcity of arable land and freshwater limits the expansion of conventional agriculture in the face of growing global protein demand. Microalgae, in turn, have a high protein content, rapid growth, and the ability to thrive in non-arable areas or in cultivation systems that utilize nutrient-rich wastewater. Furthermore, they provide high-quality proteins containing all essential amino acids. Under ideal conditions, their biomass can double in just a few hours, giving them great scalability potential. In addition to protein, microalgae are also important sources of carbohydrates, lipids, vitamins, minerals, and bioactive compounds, which enhances their nutritional value and health benefits [3,4,5,6].

Microalgae are microscopic organisms with a high potential for producing a wide range of bioactive compounds, such as proteins, lipids, carbohydrates, antioxidant compounds, and pigments. Research indicates that microalgae have an exceptionally rapid growth capacity, being able to double their biomass within a matter of hours. Furthermore, they can be cultivated in controlled environments, as photobioreactors, using only water and sunlight as their main resources [2]. This versatility, combined with high productivity, reinforces their role as emerging sources to meet the growing demand for sustainable functional ingredients. The increasing demand for natural bioactives in the food industry also reflects a global movement towards healthier ingredients with additional health benefits. Today’s consumers value products that, in addition to fulfilling basic nutritional functions, also offer antioxidant, anti-inflammatory, and neuroprotective properties [7]. In this context, the replacement of synthetic additives with natural alternatives is increasingly regarded as a market requirement in continuous expansion.

Several microalgal species are recognized as sources of bioactive compounds and nutrients. *Chlamydomonas reinhardtii*, a model green microalga, produces proteins, lipids, and bioactive polysaccharides with antimicrobial and antioxidant properties [8], though it is not yet widely commercialized for direct human consumption. *Tetraselmis* spp. are rich in proteins, lipids, and pigments and have been applied in aquaculture as well as explored for functional foods and nutraceuticals [9]. *Dunaliella salina* is notable for its high β-carotene and glycerol content, providing strong antioxidant and anti-inflammatory potential for food, nutraceutical, and cosmetic applications [10]. In comparison, *H. pluvialis* is particularly valued for its exceptional astaxanthin content, a potent antioxidant with broad applications in the pharmaceutical, nutraceutical, cosmetic, and food industries.

*H. pluvialis* is a unicellular Chlorophyceae noted as one of the richest natural sources. Biochemically, it has high contents of proteins, carbohydrates, lipids, and carotenoids, which confer important characteristics for food applications [11,12,13,14]. Its protein content (29–45%) provides a valuable ingredient for food enrichment. Carbohydrates accumulate under stress, serving as an energy reserve, with levels ranging from 15% to 74% depending on cultivation conditions. Lipid accumulation similarly increases under unfavorable conditions, with the fatty acid profile with saturated, monounsaturated, and polyunsaturated fatty acids highly strain dependent and responsive to growth parameters [14]. Astaxanthin (3,3′-dihydroxy-β-carotene-4,4′-dione), a potent natural antioxidant, has established applications in pharmaceutical, nutraceutical, cosmetic, and food and beverage industries [11,14].

**Figure 2:**
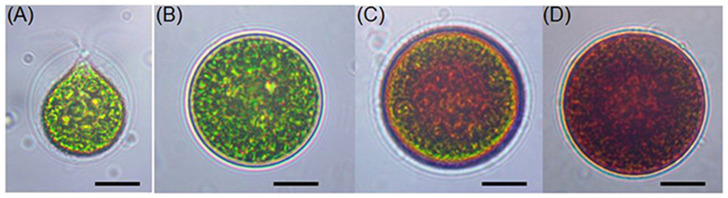
Light microscopic images of *H. pluvialis* cells in the life cycle. (**A**) Green vegetative motile cell; (**B**) green vegetative palmella cell; (**C**) astaxanthin-accumulating palmella cell in transition to aplanospore; (**D**) astaxanthin-accumulated aplanospore cell. Scale bar: 10 μm. Source: Shah et al., 2016 [11].

Currently, the production of *H. pluvialis* is primarily associated with astaxanthin extraction, which has already been approved for human consumption in countries such as the United States and the European Union. However, astaxanthin production from *H. pluvialis* still involves high costs [15]. Moreover, after extraction, the biomass residues are generally discarded, even though recent studies highlight their potential in fermentation, animal feed, and biofuel production. The application of these residues, or even the whole biomass, in the field of human nutrition remains little explored [16]. Astaxanthin has been recognized for its remarkable antioxidant capacity, greatly exceeding that of other carotenoids and commonly used synthetic antioxidants [17]. Beyond its function as a natural pigment, astaxanthin has been linked to the prevention of oxidative stress-related conditions, including cardiovascular, neurodegenerative, and inflammatory disorders, underscoring its potential as a valuable ingredient in functional foods. The unique life cycle of *H. pluvialis* (Figure 2) is also attractive from a biotechnological perspective. During the vegetative phase, the microalga accumulates green biomass; however, under adverse conditions, such as high light intensity or nutrient limitation, it enters the cyst stage and initiates the biosynthesis of astaxanthin in large quantities [18,19]. This adaptive mechanism represents a unique opportunity to explore cultivation strategies that optimize production at an industrial scale.

Several recent studies have aimed to enhance astaxanthin productivity through innovative cultivation strategies, including mixotrophy with agro-industrial residues and the application of physical stimuli, such as mechanostimulation [20]. These innovations increase biomass and bioactive yields, but despite these advances, challenges related to astaxanthin extraction and purification persist, since conventional methods present economic and environmental limitations, while emerging technologies still lack validation at a large scale [21]. Furthermore, regulatory issues and consumer acceptance need to be overcome for astaxanthin derived from *H. pluvialis* to gain a larger share in the global natural bioactive market.

This review aims to consolidate the current knowledge on *H. pluvialis* as a source of bioactives for food applications. Biological and physiological aspects, cultivation strategies, extraction methods, and potential applications in the food industry are discussed. Additionally, the review seeks to identify research gaps and future challenges, providing a critical and integrative perspective on the role of this microalga in the development of functional ingredients.

## 2. Biology and Physiology of *Haematococcus pluvialis*

### 2.1. Taxonomy

*H. pluvialis* is a unicellular, spherical, green, biflagellate, oleaginous cell [22]. It belongs to the class Chlorophyceae, order Volvocales, and family *Haematococcaseae* [23]. It is also known as *Haematococcus lacustris* or *Sphaerella lacustris* [11]. It was first mentioned in 1844 by J. Von Flotow, and later, in 1899, Tracy Elliot Hazen described its biology and life cycle [24,25]. It is commonly found in temporary water bodies, such as rain pools, artificial pools, natural and artificial ponds, and birdbaths [26,27].

**Figure 1:**
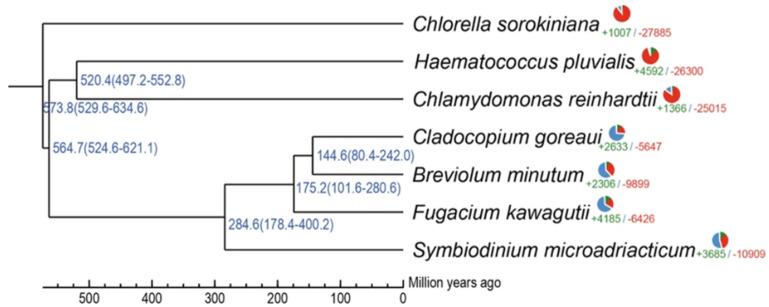
Phylogeny and gene family analysis of seven representative microalgal species. The figure was adapted from Bian et al., 2023 [28].

Figure 1 shows the phylogenetic and gene family analyses, placing *H. pluvialis* close to *Chlamydomonas reinhardtii* and *Chlorella sorokiniana*. This proximity reflects the evolutionary relationship among these species of green flagellate microalgae and demonstrates that these lineages diverged from more distant groups, such as the symbiotic dinoflagellates (*Cladocopium goreaui*, *Breviolum minutum*, *Fugacium kawagutii*, and *Symbiodinium microadriaticum*), more than 400 million years ago. [28].

### 2.2. Morphology and Life Cycle

The cellular structure of *H. pluvialis* resembles that of most other unicellular green volvocalean algae. Its life cycle consists of four distinct cellular morphologies: macrozooids (zoospores), microzooids, palmella, and hematocysts (aplanospores) [24,29,30]. The first three are known as the “green vegetative phase”, while the fourth is known as the “immobile red phase, encysted and enriched with astaxanthin”. During its growth stages, it exhibits both motile and non-motile forms [11].

In the macrozooid (zoospore) phase, the cells are spherical, ellipsoidal, or pear-shaped (Figure 2A). Their cellular structure includes a cup-shaped chloroplast with numerous and dispersed pyrenoids, frequently numerous contractile vacuoles seemingly distributed irregularly near the protoplast surface, a nucleus, and two flagella of equal length emerging from the anterior papilla that penetrate the cellulose wall. The structure is characterized by a strongly thickened, gelatinous cell wall, usually connected to the protoplast by simple or branched filaments [31].

At this stage, the cells range from 8 to 20 μm in length and exhibit rapid growth under favorable conditions. Macrozooids can divide into 2–32 daughter cells by mitosis [32]. Under unfavorable environmental or cultural conditions, macrozooids begin to lose their flagella, increase in cell size, and develop into the palmella stage (Figure 2C). In this phase, they become immobile and transform into resting vegetative cells (Figure 2B) [33].

With continued environmental stress, such as nutrient deprivation, high light intensity, or high salinity, cell division ceases, and the palmella transforms into asexual “aplanospores” (Figure 2D). At this stage, the cells contain two distinct structures: a thick, rigid trilaminar sheath and a secondary cell wall composed of acetolysis-resistant material. At this stage, the cells are more resistant to extreme environmental conditions [11,34]. This general process is called “encystment”, during which the protoplast presents a pronounced red coloration, attributed to a secondary carotenoid, astaxanthin [30]. Astaxanthin is accumulated in lipid droplets deposited in the cytoplasm, resulting in the characteristic bright-red coloration of these cells [33]. Brinda et al. (2004) reported that some *H. pluvialis* strains are capable of accumulating astaxanthin without forming aplanospores [35]. Once environmental or cultural conditions return to the optimal state, the red aplanospores germinate to form flagellated zoospores, initiating a new vegetative growth cycle [11].

### 2.3. Growth Conditions and Environmental Influence

The growing interest in this microalga as a source of natural astaxanthin has led to numerous studies aimed at optimizing its growth conditions. Various methods for cultivating and producing astaxanthin under photoautotrophic, heterotrophic, and mixotrophic conditions, both in closed systems, open raceway ponds, or closed photobioreactors using batch or fed-batch approaches, have been reported [36,37,38].

Photoautotrophic culture conditions require light, CO_2_, water, and nutrients. Light serves as an energy source, while the inorganic compounds mainly provide carbon and nitrogen for producing algal biomass rich in lipids, proteins, and carbohydrates [39,40]. To supply nitrogen needs, sodium nitrate, ammonium nitrate, and urea are being studied as possible sources of this nutrient [41]. This condition is primarily implemented in open raceway ponds or closed photobioreactors. Typical photobioreactors used for its cultivation could be tubular, bubble column, and air-lift photobioreactors [11]. Under heterotrophic culture conditions, *Haematococcus* is supplied with organic compounds, such as sodium acetate, sodium gluconate, and glycerol, that serve as sources of carbon and energy for growth and secondary metabolite synthesis in the absence of light to enhance cellular productivity [14,42].

Nevertheless, this technique is not truly suitable for astaxanthin production in *H. pluvialis*, since astaxanthin is a light-dependent carotenoid [43]. On the other hand, in the mixotrophic mode, the use of organic and inorganic sources of carbon and energy, as acetate-supplemented media, has been successful in enhancing *H. pluvialis* growth and astaxanthin production [44]. Recent advances in *H. pluvialis* cultivation for astaxanthin production include a two-stage mixotrophic culture system [45]. Boussiba and Vonshak (1991) were able to establish conditions that provide optimal photoautotrophic vegetative growth, with a specific growth rate of 0.054 h^−1^ (one of the highest reported), corresponding to a doubling time of 13 h [34].

In addition to the cultivation process, the induction of carotenoid synthesis in *H. pluvialis* is directly related to the cellular astaxanthin content and astaxanthin productivity. Studies have shown that astaxanthin accumulation is influenced by environmental factors such as light, temperature, pH, nutrient concentration, and nutritional stresses, mainly related to nitrogen deprivation [19,36,37,39,46,47,48].

## 3. Bioactive Compounds from *Haematococcus pluvialis*

*H. pluvialis* biomass is rich in essential macronutrients, positioning it as a valuable source for food applications. The cellular composition of *H. pluvialis* varies significantly according to its green and red cultivation stages and the cultivation conditions. Table 2 presents the typical composition of *H. pluvialis* at the green and red stages [12].

During the red cultivation stage of *H. pluvialis*, under continuous stress conditions such as temperature, nutrient deprivation, and light, it can accumulate up to 5.5% of its dry weight as astaxanthin [49]. During the stress period, in the astaxanthin accumulation phase, this microalgae develops a rigid cell wall composed of alginate biopolymers and cellulose-like polysaccharides. This cell wall is resistant to disruption when subjected to physical, chemical, and enzymatic treatments [50].

This species is recognized for its ability to accumulate the highest levels of astaxanthin in nature, which is why it is considered the primary natural source of astaxanthin for commercial exploitation [51]. In addition to astaxanthin, *H. pluvialis* contains a range of nutrients and bioactive compounds, such as polysaccharides, proteins, essential fatty acids, vitamins [52], and dietary fiber [53], making it a promising candidate for food enrichment.

Cultivation conditions can modulate biomass and increase levels of protein (up to 60%), carbohydrates (up to 60%), and oils (up to 70%) [54]. Most *H. pluvialis* strains have higher protein content in the green stage (29–45%) under favorable growth conditions compared to the red stage (Table 1). Its protein fraction has a balanced amino acid profile, including essential amino acids, which gives the microalga a high biological value [54].

In the red stage, the protein content is around 21–23% of the cellular content [21], and the total amino acid content is approximately 10 mg/100 mg, composed of aspartic acid, glutamic acid, alanine, and leucine, with 46% being essential amino acids [50]. Bassani et al. (2025) quantified in *H. pluvialis* biomass an amino acid content of 34.83 mg/100 mg, identifying 13 amino acids, with valine, isoleucine, and glutamic acid being the most abundant [55].

Regarding carbohydrate content, the highest accumulation of this macronutrient occurs in the red stage, which includes polysaccharides such as starch, cellulose, and glucans. These polysaccharides contribute to the cellular structure and possess functional properties [14]. Starch aids in cellular protection, allowing the cell to survive prolonged stress. The content increases from 15 to 17% dry weight (dw) in the green stage to 60–74% dw in the red cyst stage. Recht et al. (2012) [13] demonstrated that total carbohydrate content can increase up to 63% dw during the first day of stress exposure, decrease to 41% dw the following day, and remain at this level until the end of cultivation.

Dos Santos et al. (2017) [56] demonstrated that, although carbohydrates are essential for cellular metabolism, limited research has addressed their synthesis in *H. pluvialis.* They investigated the influence of pH in the range of 6.0 to 7.2 on vegetative growth and the biochemical composition of *H. pluvialis* and found that pH variation in cultures can be problematic for *H. pluvialis,* while maintaining pH within a narrow range resulted in better growth conditions and, consequently, improved biomass composition.

Lipid content can range from 20 to 25% in the green stage, while in the red stage, cells can generate up to 40% of their cellular weight as cytoplasmic lipid droplets, along with a significant amount of secondary metabolites, including up to 4% of the carotenoid astaxanthin [21]. Astaxanthin accumulation in *H. pluvialis* is directly related to lipid accumulation, which in turn depends on de novo fatty acid synthesis. The formation of oil globules in *Haematococcus* is associated with the endoplasmic reticulum membranes. Under nitrate deprivation, astaxanthin and fatty acids can account for up to 4% and 40%, respectively, of the cell’s dry mass [57]. Because of its high lipid content, research has shown good results in the production of biodiesel from *Haematococcus pluvialis* biomass [58].

The total fatty acid profile of *H. pluvialis*, which includes palmitic, linoleic, and linolenic acids, is flexible and can vary depending on the strain. This variation can be attributed to several factors, comprising culture conditions, strain origin, and environmental stresses, such as nitrogen limitation, high salinity, phosphorus deficiency, extreme temperatures, and high light intensity. Furthermore, studies indicate that the highest lipid content in *H. pluvialis* is achieved when the microalga is cultivated under nutrient-depleted conditions [13,14,21]. According to Hagen et al. [33], the most effective factor inducing lipid accumulation in the cell is nitrogen limitation.

Researchers have demonstrated that *H. pluvialis* cells increased their lipid content from 15.61% to 34.85% of cell dry weight when exposed to continuous high light intensity in nitrogen-sufficient medium or reached 34.85% and 32.99% of cell dry weight when subjected to continuous high light intensity under nitrogen-depleted conditions. The fatty acid profile, reported by Damiani et al. (2010) [59], indicated that the main components were palmitic, stearic, oleic, linoleic, linolenic, and linoleic acids [60]. Besides the macronutrients mentioned, the microalgae is also a source of vitamins such as B12, K, C, and E, as well as minerals, which contribute to the enhancement of its nutritional and bioactive value [52,61].

The presence of carotenoids, in addition to astaxanthin (such as lutein and β-carotene), also contributes to the antioxidant and pigmentary profile of the biomass [62]. Carotenoid content changes during cellular transitions, increasing from 0.5% (dry weight) in the green phase to 2–5% (dry weight) in the red phase. Lutein, with a content of 70–80%, is the main carotenoid present in green cells. The second most abundant component is β-carotene (16.7% dry weight), while the amounts of violaxanthin and neoxanthin are 12.5% and 8.3% (dry weight), respectively. These carotenoids are either absent or present in very low amounts in red-phase cells. Another pigment found only in green cells is chlorophyll, with a content of 1.5–2% dry weight [11,14].

The most important carotenoid obtained from *H. pluvialis* is astaxanthin, which is accumulated within the cell only during the red phase. Its content can reach up to 80–99% of the total carotenoids [63]. Astaxanthin (3,3′-dihydroxy-β-carotene-4,4′-dione) is a bright red secondary carotenoid belonging to the same family as lycopene, lutein, and β-carotene. It contains two chiral centers and can exist as three different stereoisomers: (3S,3′S), (3R,3′S), and (3R,3′R). A 1:2:1 ratio of these isomers is obtained during chemical synthesis of the compound. *H. pluvialis* is capable of predominantly biosynthesizing the (3S,3′S) stereoisomer, which is the most valuable [64]. Astaxanthin synthesis in *H. pluvialis* is directly correlated with the deposition of cellular reserves in lipid droplets under conditions of cellular stress [65].

Astaxanthin possesses one of the strongest known antioxidant effects; due to its unique structure, it is capable of crossing biological membranes and acting as an antioxidant by neutralizing and stabilizing free radicals [66]. It is commercially available mainly as dietary supplements, oils, or dried aplanospores. However, the cell walls of aplanospores need to be disrupted to improve digestibility. Oils containing astaxanthin are not organoleptically attractive due to the characteristic taste and odor of algae. Nonetheless, in recent years, there has been increasing research on the incorporation of astaxanthin into foods and animal feeds [14].

## 4. Cultivation Strategies for Enhanced Bioactive Production

### 4.1. Open Ponds vs. Photobioreactors

The cultivation of *H. pluvialis* can be conducted in open systems, such as ponds, and closed systems, such as photobioreactors. Open ponds have low installation and operation costs; however, they present significant disadvantages, such as high susceptibility to contamination by undesirable microorganisms, pollution, and complete dependence on climatic factors, which ultimately make cultivation less efficient and less productive [67].

On the other hand, photobioreactors provide a fully controlled cultivation environment, allowing precise adjustments of essential parameters such as pH, temperature, light intensity and spectrum, and nutrient concentrations. This control results in a significant increase in biomass productivity and in a more efficient accumulation of biocompounds of interest, such as astaxanthin, although the initial cost and operating expenses are higher [68].

Intermediate alternatives, such as hybrid systems, have been used as a solution to reduce costs in large-scale production, combining the growth phase in photobioreactors with the accumulation phase of biocompounds in open tanks [69].

### 4.2. Environmental Stress Factors

Astaxanthin is produced as a defense mechanism by the microalga against environmental stress. Its natural content is relatively low, yet of great industrial interest, as it has better antioxidant properties than its synthetic form. The accumulation of this biocompound can therefore be induced under adverse conditions that activate the pathways involved in biosynthesis [70]. Light intensity and spectrum are among the most tested and effective conditions to induce this stress. Blue wavelength increases oxidative stress, which drives the microalga to produce astaxanthin to protect itself against possible cellular damage [71].

In addition to astaxanthin, *H. pluvialis* also synthesizes and accumulates another carotenoid of great relevance for food applications, canthaxanthin. This xanthophyll, with a reddish-orange coloration and structurally related to astaxanthin, is widely used as a natural colorant and also as a feed additive in animal nutrition. This carotenoid exhibits antioxidant properties that contribute to the oxidative stability of foods and may act as a protective agent against cellular damage associated with oxidative stress [72]. Another noteworthy carotenoid is β-carotene, a direct precursor of astaxanthin, which, in addition to playing an essential role in the biosynthetic pathway, also has its own commercial interest as a natural colorant and a source of provitamin A, further reinforcing the biotechnological potential of the species [73].

Nutrient deprivation, such as nitrogen limitation, is another highly effective strategy for astaxanthin production, as its absence redirects energy and metabolic precursors toward carotenoid biosynthesis instead of cell growth. To optimize this production, it is essential to adjust nitrogen concentration so that deprivation stimulates biocompound synthesis without compromising the overall culture development. Therefore, understanding the cultivation objective is essential to defining the best strategy [41].

Under nitrogen limitation and high light intensity, the microalga also accumulates large amounts of neutral lipids, such as triacylglycerols, which are often associated with astaxanthin in the form of esters, thereby increasing the biotechnological interest in fatty acids for applications as functional ingredients [59].

Increased salinity, in turn, generates osmotic stress that triggers astaxanthin synthesis. This strategy is considered simple and low-cost, as it can be carried out by the controlled addition of salts to the culture medium. Salt stress acts as a signal to activate carotenoid biosynthetic pathways [74].

Temperature is also an important factor that affects biomass growth and lipid production, the latter being an important component with various industrial applications. Studies have shown that the optimal cultivation temperature to achieve a balance between biomass yield and lipid productivity is 20 °C [20].

Thus, although astaxanthin remains the main target of interest and the most extensively studied compound in the literature, there is growing evidence that stress conditions and environmental changes in cultivation also favor the production of other bioactives. This set of metabolic responses reinforces the potential of *H. pluvialis* as a producer of diverse high-value molecules, thereby broadening its application prospects.

### 4.3. Biotechnological Approaches (Genetic Engineering, Omics Studies)

In the field of biotechnology, significant advances have been achieved in optimizing the production of biocompounds of industrial interest. One example is astaxanthin, whose biosynthesis has been the focus of genetic engineering strategies. A detailed understanding of the metabolic pathways involved and the genes responsible for the biosynthesis of this biocompound, such as rtB (phytoene synthase) and crtO (β-caroteno cetolase), enables manipulations that increase productivity [75]. The use of precise gene-editing tools, such as the CRISPR-Cas system, has allowed specific modifications in genes of interest, generating strains with higher carotenoid accumulation, as well as the silencing of negative regulators using techniques such as RNA interference (RNAi) [76].

Studies have shown, for instance, that the overexpression of a modified pds gene in transgenic *H. pluvialis* strains resulted in up to a 36% increase in astaxanthin production compared to the wild-type strain after 48 h of light induction [75]. In addition, overexpression and silencing approaches have targeted genes such as lcyB, bkt/crtW, and isoprenoid flux regulators, which affect not only the balance between astaxanthin and β-carotene but also the degree of esterification and the storage of lipid molecules [73].

Beyond direct genetic manipulation, omics studies have revolutionized the understanding of *H. pluvialis* biology. Transcriptomic analyses under different stress conditions have revealed gene co-expression networks and identified transcription factors associated with the carotenoid pathway and the formation of the secondary cell wall [73].

A recent advance was the release of a dataset comprising 96 RNA-seq samples, covering different treatments and experimental time points, which enabled gene co-expression analyses and provided a robust public resource for the identification of regulators associated with carotenoid biosynthesis. These data represent a valuable resource for a better understanding of the molecular mechanisms that govern critical processes in *H. pluvialis* [77].

In addition, metabolomic approaches have demonstrated that exposure to environmental stresses triggers a profound reprogramming of central metabolism, redirecting carbon flux toward the formation of triacylglycerols and astaxanthin esters, while integrated metabolomic and physiological studies have been elucidating control points that can be exploited in two-stage cultivation strategies [78].

Proteomic studies in *H. pluvialis* have revealed that phosphorylation acts as an important regulatory layer over pathways related to cell division, nutrient partitioning, and acetate metabolism, highlighting protein targets with potential for strain engineering. In addition, integrated transcriptomic and proteomic analyses under blue light have supported the existence of post-transcriptional control points in the carotenoid biosynthetic pathway [73].

These studies allow the understanding of how the microalga responds to environmental stimuli at the molecular level and reveal new potential targets for biotechnological intervention [67,76]. The integration of transcriptomic data with metabolomics and proteomics provides a broader and more refined view of the regulatory pathways involved, supporting the development of more productive strains [76].

The optimization of cultivation conditions, combined with the strategic application of stress factors and the use of advanced biotechnological tools, offers a promising route for enhancing the production of biocompounds in *H. pluvialis* [79]. Nevertheless, the molecular mechanisms underlying these biosynthetic processes remain only partially understood, with important gaps still to be filled regarding their regulatory pathways [70].

## 5. Extraction and Purification of Bioactives

### 5.1. Conventional Extraction Methods

The extraction of astaxanthin and other bioactive compounds from *H. pluvialis* has traditionally been conducted using organic solvents such as acetone, ethanol, methanol, and hexane. These methods offer the advantages of low initial cost and relative operational simplicity and are widely employed at both laboratory and semi-industrial scales [21]. These solvents often exhibit toxicity and low selectivity, in addition to generating waste that requires proper disposal. The thick cell wall of *H. pluvialis* represents a significant barrier, necessitating additional cell disruption steps such as bead milling, high-pressure homogenization, or freeze-drying to enhance extraction efficiency [11]. Although effective, these conventional methods are increasingly being questioned in terms of their sustainability and safety for food applications. Several studies have investigated conventional astaxanthin extraction methods from *H. pluvialis*, primarily using organic solvents and various pretreatment techniques to break the microalga’s resistant cell wall.

Pitacco et al. (2022) [80] They highlight the effective use of various traditional organic solvents ranging from the most toxic to the safer ones in the astaxanthin recovery process. Bauer and Minceva (2019) [81] developed an innovative approach using germinated cells (zoospores) in a continuous liquid–liquid chromatography system, bypassing conventional steps such as drying and cell disruption, and achieving yields of up to 85% with ethyl acetate. The rigid cell wall of *H. pluvialis* poses a significant challenge to efficient extraction. Irshad et al. (2019) [82] employed planetary ball milling as a pretreatment, followed by extraction using either conventional solvents or supercritical CO_2_, obtaining 31.4 mg/g of astaxanthin along with high antioxidant activity.

Ruen-ngam et al. (2010) [83] compared different solvents and found that acetone yielded the highest astaxanthin recovery compared to methanol, ethanol, and acetonitrile. Another study evaluated four extraction methods: hydrochloric acid followed by acetone (HCl-ACE), a hexane/isopropanol mixture, a two-step extraction (methanol + acetone), and soybean oil extraction. The HCl-ACE method showed the best results, with an oil yield of 33.3% and astaxanthin content of 19.8 mg/g of cells [84]. Although conventional extraction methods using organic solvents are widely employed due to their simplicity and lower cost, they present significant limitations related to toxicity, selectivity, and environmental impact. The need for pretreatment steps to break the cell wall of *H. pluvialis* increases the complexity of the process and may compromise the quality of the final product. These challenges have driven the search for more sustainable and efficient alternatives, such as green extraction technologies, which are discussed in the following section.

Recently, a new study explored the selective extraction of proteins, lipids, and carbohydrates from *H. pluvialis* using a novel temperature-responsive microemulsion (ME) composed of ionic liquid-based deep eutectic solvents (DES). Results demonstrated that the DES-based ME achieved high extraction efficiencies, yielding 313.6 mg/g for lipids, 150.1 mg/g for proteins, and 26.98 mg/g for carbohydrates. Post-extraction, cooling-enabled demulsification facilitated the selective separation of these biocomponents: lipids (and carotenoids) partitioned into the DES-rich phase, while proteins migrated to the water-rich phase. This methodology represents a significant advancement for the comprehensive utilization of *H. pluvialis,* overcoming the limitations of traditional methods in simultaneously separating hydrophobic and hydrophilic compounds [85].

### 5.2. Emerging Green Technologies

The use of sustainable and safe methods for extracting bioactive compounds, such as astaxanthin, has been growing and gaining recognition as emerging green technologies. These approaches minimize the use of toxic solvents, reduce environmental impact, and can also enhance process efficiency. The use of supercritical carbon dioxide (SC-CO_2_) has emerged as a promising alternative to traditional organic solvent extraction. This technology enables the selective extraction of bioactive compounds without leaving toxic residues and preserves the integrity of the target molecules due to its operation at moderate temperatures. Combining SC-CO_2_ with co-solvents such as ethanol significantly improves extraction efficiency [11].

Another widely used method is ultrasound-assisted extraction, which employs high-frequency sound waves to generate cavitation, promoting cell wall disruption and facilitating the release of intracellular compounds. This technique shortens extraction time, minimizes solvent use, and is compatible with green solvents such as ethanol or ionic liquids. Studies have shown that such methods can increase the extraction of bioactive compounds by up to 30% compared to conventional techniques [82]. Microwave-assisted extraction is also highlighted as a non-thermal technology that rapidly heats the biomass, causing expansion and cell rupture, thereby facilitating compound extraction. This method is efficient, fast, and requires little to no solvent. When combined with appropriate pretreatment steps, it has the potential to overcome the limitations imposed by the thick cell wall of the microalga [21].

Ionic liquids and natural deep eutectic solvents (NADES) have also been studied and applied, as they exhibit low toxicity and high biodegradability. These solvents offer high selectivity for the extraction of polar compounds, such as carotenoids. Although still in the early stages of application, recent studies indicate their effectiveness in astaxanthin extraction, achieving competitive yields with reduced environmental impact [80].

### 5.3. Comparative Efficiency and Sustainability

The different methods show promising results in terms of efficiency and sustainability; however, some are noteworthy, as shown in Table 2. This table presents a comparison between conventional and emerging methods regarding extraction efficiency, processing time, solvent toxicity, industrial scalability, and overall sustainability.

**Table 2:** Comparison between conventional and emerging extraction methods for astaxanthin. n.d: no data.

Extraction Method	Compound Yield (mg/g)	Toxicity	Advantages	Disadvantages	Industrial Scalability	Cost	Ref.
Organic Solvents (Acetone, Ethanol, Hexane)	Astaxanthin 15–31.4	Use of toxic solvents, low selectivity	Simple operation	Requires cell disruption pretreatments	Widely used in labs and semi-industrial scale	Low initial cost	[21]
Supercritical CO_2_	Astaxanthin Up to 40	Non-toxic solvent	High selectivity, minimal residue, mild temperature	Requires high pressure	Used in industries and on a lab scale	High equipment cost	[11]
Ultrasound-Assisted Extraction	Astaxanthin 30–35	Can be toxic	Fast, reduced solvent usage, efficient when combined with pretreatment	Requires specific equipment, possible localized heating	Pilot and semi-industrial scales	Low cost	[82]
Microwave-Assisted Extraction	Astaxanthin 28–32	Nontoxic	Rapid process, preserves bioactives, low energy consumption	Needs specialized equipment, critical temperature control	Applied at lab or pilot scale	Low cost	[21]
Ionic Liquids/Natural Deep Eutectic Solvents	Astaxanthin 25–38	Depends on the composition of the solution	High selectivity, biodegradable, and recyclable solvents	High viscosity and limited availability	Medium cost	High cost	[80]

The bastaxanthin extraction methodologies reveal a complex interplay of yield, toxicity, scalability, and cost, all critical for optimizing industrial production. While organic solvents offer low initial cost and simple operation, their use of toxic solvents, low selectivity, and the necessity for cell disruption pretreatments present significant environmental and processing challenges, despite their widespread use at semi-industrial scales [21]. In contrast, supercritical CO_2_ (scCO_2_) extraction stands out for its high selectivity, non-toxic solvent profile, and minimal residue, achieving yields of up to 40 mg/g. However, its high equipment cost and requirement for high pressure limit its industrial scalability, primarily to lab-scale applications [11]. Ultrasound-assisted extraction (UAE) provides a fast process with reduced solvent usage and can be efficient when combined with pretreatment, offering a low-cost solution applicable at pilot and semi-industrial scales. A notable concern, however, is its potential toxicity when certain solvents are employed, as well as the need for specific equipment that might lead to localized heating [82]. Microwave-assisted extraction (MAE) is characterized by its rapid process, preservation of bioactives, and low energy consumption, with a non-toxic profile and low cost. Yet, it demands specialized equipment and critical temperature control, typically applied at lab or pilot scale [21]. Finally, ionic liquids and natural deep eutectic solvents represent an emerging green alternative, boasting high selectivity, biodegradability, and recyclability. While offering medium to high yields (25–38 mg/g), their high viscosity, limited availability, and higher cost (medium to high, depending on the specific solvent) pose hurdles for broad industrial adoption [80].

## 6. Applications in the Food Industry

### 6.1. Functional Ingredients (Supplements, Fortification, Beverages)

Microalgae have been consumed as a dietary component for thousands of years, particularly in traditional cultures. In the past decades, the rising recognition of the interplay between diet and health has intensified consumer demand for functional foods. This trend has positioned microalgae as promising ingredients due to their rich profile of bioactive compounds such as pigments, polyunsaturated fatty acids, and polysaccharides, which can contribute to nutritional quality and physiological benefits [86]. Among these, *H. pluvialis* has gained attention as a versatile ingredient, with applications ranging from bakery products to innovative food systems.

The current interest in applying *H. pluvialis* in the food industry is related to the content of carotenoids, proteins, lutein, and fatty acids, which can provide and enhance several properties in food [87]. In bakery products, it can enhance antioxidant capacity, modulate glycemic response, and contribute to appealing color and texture [51]. In beverages, it enriches protein and beneficial fatty acids while promoting the growth of probiotic bacteria [88]. In meat analogs, it improves color, texture, and aroma, and in advanced emulsified systems, it supports the structural stability suitable for 3D food printing and fat replacement [89].

For example, *H. pluvialis* has been incorporated into whole grain cookies, resulting in higher antioxidant capacity and modulation of the glycemic response. The addition also produced a reddish coloration and a softer texture, while sensory acceptability remained unaffected [87]. The incorporation of this microalga can simultaneously strengthen the nutritional profile of bakery products and contribute to sensory characteristics that promote consumer acceptance [87]. A similar functional and technological profile is observed in the development of non-dairy fermented beverages, such as water kefir, where the incorporation of *H. pluvialis* biomass increases protein and beneficial fatty acid contents while promoting the growth of probiotic lactic acid bacteria, such as *Liquorilactobacillus nagelii* and *Lactococcus lactis*. In addition to its potential prebiotic effect, the microalga enhances the microbiological functionality of beverages designed for specific consumer groups, such as vegans and lactose-intolerant individuals [88].

In the field of plant-based meat analogs, the incorporation of *H. pluvialis* into plant protein extrudates has proven effective in enhancing color, bringing it closer to the characteristic red of beef. At the same time, the biomass contributes to a softer texture and enriches the aromatic profile with volatile compounds, such as alcohols, esters, and terpenes, which impart herbal and cereal-like notes, thereby increasing the sensory appeal of these products [89]. Beyond conventional applications, the potential of *H. pluvialis* has been explored through the valorization of its residues in the development of Pickering-type emulsified gels. These systems display rheological properties compatible with three-dimensional food printing, ensuring both structural stability and shape fidelity. Such characteristics allow their incorporation as fat replacers in desserts or personalized formulations, contributing to reduced caloric content while improving nutritional quality [90]. Despite this promising scenario, its commercial application still faces challenges related to production costs, compound stability during processing, and sensory acceptability. To overcome these limitations, strategies such as encapsulation, valorization of post-extraction residues, and the adoption of emerging technologies have been investigated, offering avenues to enhance its feasibility and competitiveness in the food industry [88,90].

### 6.2. Natural Food Colorants

Color represents a key attribute in food products, directly influencing consumer acceptability and perceived attractiveness. Driven by consumer demand, synthetic colorants are increasingly being replaced with naturally derived alternatives, which not only provide visual appeal but also confer health-related benefits such as antioxidant, anticancer, and anti-inflammatory activities [91]. Astaxanthin is a lipid-soluble, orange-red carotenoid that naturally serves as a coloring agent in fish and animal feeds and is employed in the food and cosmetics sectors [16]. *H. pluvialis* contains 17 identified compounds, with astaxanthin isomers representing the predominant carotenoids, accounting for approximately 77% [92].

Considering applications as colorants, pea protein-based meat analogs were formulated by incorporating *H. pluvialis* residue (HPR) through high moisture extrusion. Blends containing 10 to 40 g/100 g of HPR (dry basis) were successfully processed at 50 g/100 g moisture content. The intrinsic reddish pigmentation of HPR imparted a visual resemblance to dried red meat. Moreover, HPR addition significantly modified the microstructure, loosening the layered and fibrous organization of the extrudates and thereby improving their textural attributes. The measurements of the fiber index were conducted. The fibrous degree was presented using the ratio of F_V_ (crosswise shear force, F_V_) to F_L_ (lengthwise shear force, F_L_). The highest fibrous index (1.28 ± 0.05) was obtained at 10 g/100 g HPR incorporation. The authors reported that the improved texture of meat analogs was primarily attributed to the higher free water content and the increased β-sheet structure of the extrudates following HPR addition [93].

The incorporation of *H. pluvialis* (HP, 0.25 to 1.25%) as a natural colorant during high moisture extrusion (50% moisture) was investigated in soy protein-based meat analogs. The stability of the HP-induced meat-like color was assessed under light exposure, freeze freeze–thaw cycles, frozen storage, and varying cooking conditions. At low inclusion levels, HP promoted the formation of flexible and disordered regions in the protein secondary structure, whereas excessive addition hindered protein cross-linking. The highest degree of texturization was obtained with 0.75% HP. Sensory analysis indicated that 1% HP imparted a color comparable to fresh beef sirloin, while 0.25% HP produced a color more similar to fresh pork loin. The results suggest that HP is a promising natural colorant for HMMA production [94].

### 6.3. Antioxidant and Anti-Inflammatory Properties in Food Systems

Recognized for its extensive health benefits, astaxanthin is a particularly potent antioxidant that helps protect cells from oxidative stress and damage [12]. From a technological standpoint, the thermal resistance of *H. pluvialis*, with degradation occurring only above 250 °C, indicates that its bioactive compounds are preserved under common food processing conditions, such as baking and extrusion [51,92]. Encapsulation of these compounds also represents a strategy to protect them from degradation under high-temperature conditions [95]. Compared with other microalgae or natural ingredients, with this combination of high thermal stability, substantial lipid content (up to 41%, predominantly mono- and polyunsaturated fatty acids), and significant pigment fraction, *H. pluvialis* is particularly suitable for functional formulations targeting cardiovascular health, immune modulation, and cellular protection [91,93].

Astaxanthin has been shown to effectively substitute synthetic antioxidants such as butylated hydroxytoluene (BHT) in meat products, particularly sausages. Its incorporation enhances oxidative stability, providing a comparable reduction in malondialdehyde formation (a marker of lipid oxidation) to that achieved with BHT, while simultaneously addressing health concerns associated with the potential carcinogenicity of synthetic antioxidants [96]. Another example was conducted by applying astaxanthin to replace synthetic antioxidants in emulsion-type sausages, where its incorporation improved antioxidant activity and sensory attributes (color) during refrigerated storage, thereby demonstrating its potential as a multifunctional additive [97].

Marine-derived microalgae rich in astaxanthin have been incorporated into wholemeal cookies, demonstrating improved nutritional and functional properties. Substitution with 5–15% astaxanthin reduced glucose release during in vitro digestion but also increased total phenolic content and antioxidant capacity across wheat, barley, and oat formulations. The results show the potential use of microalgae to enhance the bioactive compounds and lower the glycemic response of wholemeal flour cookies [53].

### 6.4. Stability of H. pluvialis Bioproducts

The stability of *H. pluvialis* biomass and its derived bioproducts is highly dependent on storage and processing conditions. Post spray drying, the biomass exhibits minimal astaxanthin degradation when stored under refrigeration (−21 °C) in an inert atmosphere (nitrogen), with losses below 10% over a 9-week period. Conversely, exposure to light and oxygen significantly accelerates degradation, resulting in losses ranging from 30% to 70%, depending on the intensity and duration of exposure [54].

Astaxanthin is particularly sensitive to heat, oxygen, UV radiation, and acidic pH, which limits its stability during food processing and can result in substantial degradation. For instance, spray-drying can lead to astaxanthin losses of 30–40%, highlighting the need for careful handling. To preserve its integrity and bioactivity, storing *H. pluvialis* powder or other natural astaxanthin products under vacuum, in the dark, and at temperatures below 4 °C has been identified as the most practical and economical approach [93,96].

Given the sensitivity of astaxanthin to heat, light, oxygen, and acidic conditions, protective strategies are necessary to maintain its bioactivity during processing and storage. One effective approach is encapsulation, in which astaxanthin is incorporated into a carrier material that shields it from environmental stressors. Food-grade biopolymers such as carbohydrates, gums, proteins, or lipids are commonly used as encapsulating matrices. Various micro- and nanoencapsulation techniques have been developed, including spray-drying, spray-chilling, extrusion coating, liposome entrapment, complex coacervation, and nanoprecipitation. These methods aim to reduce degradation, improve thermal and photostability, and enhance bioavailability, providing a practical solution to overcome the limitations of astaxanthin’s inherent instability [98].

Another alternative is microencapsulation to improve the stability and bioavailability of astaxanthin. The spray dryer was applied to astaxanthin using carriers such as maltodextrin, gum arabic, or proteins that achieve encapsulation efficiencies of 60–94%, significantly enhancing thermal and photostability [99]. Nanoemulsions and lipid-based nanodispersions have reached 80–90% encapsulation efficiencies, offering superior stability in aqueous systems and improved bioavailability [100].

### 6.5. Evidence from In Vitro, In Vivo, and Clinical Studies

Beyond conventional applications, such as in animal feeds, its rich composition has driven the incorporation of *H. pluvialis* into diverse matrices and formulations, serving functional and technological purposes. Recently, *H. pluvialis* was employed as a fishmeal substitute in the diets of Macrobrachium amazonicum post-larvae at 50% and 100% replacement levels. Both diets yielded positive results; however, complete substitution with the microalga promoted particularly favorable outcomes, including enhanced protein productivity, increased length and weight gain, and higher survival rates [101].

Another study investigated *H. pluvialis* as a natural source of astaxanthin for clownfish diets by testing the optimal extraction method and assessing the pigmentation performance of crude extracted astaxanthin. Direct application of *H. pluvialis* powder resulted in low astaxanthin utilization. Among the extraction methods tested, the HCl–acetone approach yielded the highest concentration (21.99  ±  0.52 mg/g cell). Feeding trials with the extracted astaxanthin showed superior pigmentation compared to synthetic astaxanthin, with significant enhancement of red coloration at 200–400 mg/kg after six weeks. Overall, *H. pluvialis*-derived astaxanthin obtained via the HCl–acetone method effectively improved pigmentation performance in A. ocellaris, providing a cost-effective alternative to synthetic supplements [102].

A study was conducted to assess the safety of an *H. pluvialis* algal extract with high levels of astaxanthin in humans (35–69 years). Participants consumed three gelcaps per day, one with each meal. Nineteen participants received gelcaps containing the algal extract in safflower oil, providing 2 mg of astaxanthin each (treatment group), while 16 participants received gelcaps with safflower oil only (placebo group). After 8 weeks of supplementation, no significant differences were observed between the treatment and placebo groups in the analyzed parameters, except for serum calcium, total protein, and eosinophils. Although these three parameters differed statistically, the variations were minimal and clinically irrelevant. These results indicate that 6 mg of astaxanthin per day from an *H. pluvialis* algal extract can be safely consumed by healthy adults [103].

## 7. Safety and Regulatory Considerations

Among all known carotenoids, astaxanthin is considered one of the most significant due to its wide range of potential applications. The global carotenoid market reached USD 1.53 billion in 2021, with astaxanthin being the leading compound [104]. The market value of astaxanthin (natural and synthetic) reached USD 663.89 million in 2020, with this market predominantly dominated by Asia-Pacific countries. For the period from 2021 to 2027, it is forecasted that the value of astaxanthin in the Asia-Pacific market could grow by 4.5%, and the global market is expected to reach approximately USD 977.74 million by 2027. Haematococcus-based products are projected to account for USD 193.19 million by 2029. Currently, the market prices for nutraceutical-grade *H. pluvialis* astaxanthin range between USD 6000 and 7150/kg. At present, European countries, North America, and China hold a major share of the global market for *H. pluvialis*-derived astaxanthin [105].

Applications of carotenoids are found in the food, feed, cosmetic, and nutraceutical industries. Carotenoids have traditionally been used in the food and feed industries due to their color and nutritional properties. For food industry researchers, the use of microalgae as alternative ingredients may provide a strategy to enhance the nutritional quality of processed products. Hossain et al. [53] evaluated the effect of incorporating *H. pluvialis* and wholemeal flours on improving the physical and functional properties of cookies. Carotenoids are considered safe natural colorants and, as such, are added to a variety of products to enhance their color (e.g., juices, yogurts, and confectionery). In addition to providing color and nutrition, carotenoids are powerful antioxidants and are used as preservative agents to slow the oxidation process, thereby reducing food degradation and the development of off-flavors [106].

The approval for the sale of products containing microalgae or their active compounds must comply with safety and regulatory requirements, which vary between different countries and geographic regions. Companies intending to market these products must adhere to the applicable regulations, which involve submitting an application to a specific regional authority and providing scientific data and health and safety assessments [107].

In Europe, three levels of regulation are applied for the commercialization of microalgae or their components: general food safety regulation, novel food regulation, and regulations on nutritional and health claims of food products. The market inclusion of food products using whole microalgal cells or products containing microalgae must comply with food safety regulations applicable to all food products, such as Regulation (EC) Nº 178/2002 of the European Parliament and of the Council on Food Safety [108] and Regulation (EC) Nº 852/2004 on the hygiene of foodstuffs [109]. These regulations ensure public health protection by establishing common principles and providing the foundation for safeguarding human health.

Regarding novel food ingredients, EU Regulation 2015/2283 simplifies the authorization procedures within the categories of foods considered novel foods. This category includes whole insects and their parts, as well as foods derived from cell or tissue cultures from animals, plants, microorganisms, fungi, or algae, and foods of mineral origin [110].

In the United States, the Center for Food Safety and Applied Nutrition (CFSAN) of the Food and Drug Administration (FDA) is responsible for regulating food ingredients and ensuring their safety and legality. The FDA is authorized to enforce two laws applicable to microalgae-based food and feed products available in the consumer market: the Federal Food, Drug, and Cosmetic Act, introduced in 1938, which regulates all foods and food additives [111], and the Dietary Supplement Health and Education Act (DSHEA), introduced in 1994, which covers dietary ingredients and supplements. The FDA also regulates and assigns Generally Recognized as Safe (GRAS) status to products [112].

Asian countries have enjoyed the benefits of algae for centuries. Consumers are more aware of the potential of microalgae due to this rich cultural history compared to the West. In China, seaweeds, microalgae, and cyanobacteria are used as health foods and nutraceuticals, and the marketed products range from raw seaweed to tablets. These products are regulated under the 2015 Food Safety Law, enforced by the National Medical Products Administration (NMPA) (Food Safety in China). In Japan, food safety is overseen by the Minister of Health, Labor, and Welfare (MHLW) as part of the Food Safety Department, under the Pharmaceutical and Food Safety Bureau [112].

*H. pluvialis* has been approved as a dietary supplement ingredient for human consumption in the United States, Japan, and several European countries [113]. Supercritical CO_2_ extracts of *H. pluvialis* have received “novel food” status from the UK Food Standards Agency (FSA), while the US FDA has granted *H. pluvialis* astaxanthin GRAS status [12,114].

Commission Implementing Regulation (EU) 2017/2470, which establishes the list of novel foods in accordance with Regulation (EU) 2015/2283, specifies that astaxanthin-rich oleoresin from the microalgae *H. pluvialis* is authorized in dietary supplements at levels of up to 40–80 mg/day, corresponding to a maximum permitted level of 8 mg of astaxanthin per day [115].

It has been observed that, over the past decade, there has been a 150% increase in the number of companies producing algae-derived products, reflecting a rise in the development of innovative food products from algal biomass or its components [116]. The growing global demand for astaxanthin, increasing levels of research and development investment in Haematococcus, and rising preferences for microalgae-based products may potentially shape the market dynamics of Haematococcus-based products by 2030. In this context, the European Commission, through its proposed action ‘Towards a Strong and Sustainable Algae Sector’ and the EU4Algae Initiative, is at the forefront of developing the EU algae sector [117]. This initiative aims to promote sustainable production and innovation in this sector, increasing the need for updated regulatory frameworks to support the potential of algae as a food source.

### Consumer Acceptance and Market Trends

The success of microalgae as a food source depends not only on product research but also on understanding consumer preferences. Despite providing a potential novel dietary resource for advancing sustainable human nutrition, when incorporated into foods, microalgae can influence their taste, aroma, texture, and visual appearance, which, in turn, may affect product acceptability among consumers [118].

Olsen et al.’s [118] review of studies conducted on European consumers revealed that, overall, microalgae are considered acceptable as food ingredients. Nevertheless, consumers expressed a clear preference for low inclusion levels, largely due to the intense green coloration imparted to products. Furthermore, freshwater algae were generally better received in terms of flavor, whereas marine species were less favored because of their pronounced fish-like taste. A work conducted by Silva et al. [119] assessed 1499 Brazilian consumers to evaluate their knowledge about microalgae and their potential use in food products. It was observed that while most participants had limited knowledge about the benefits of microalgae, the majority were willing to consume foods containing microalgae biomass or its byproducts. Nutritional supplements, seasonings, bakery products, sauces, beverages, and dairy emerged as the most accepted categories, with 40% of respondents indicating willingness to pay a price premium. These results highlight promising opportunities for the development and commercialization of novel food products incorporating microalgae.

## 8. Future Perspectives and Challenges

The use of *H. pluvialis* in foods is linked to two fundamental pillars for the future of nutrition: sustainability and innovation. In the context of sustainability, emphasis is placed on the importance of fully utilizing the microalgal biomass and the residues resulting from astaxanthin extraction, rather than focusing solely on astaxanthin itself. Both the biomass and the post-extraction residues have the potential to be incorporated into food formulations, transforming what would otherwise be discarded into valuable new resources. This strategy not only reduces waste but also strengthens more efficient production chains aligned with the principles of the bioeconomy.

Advances in biotechnology and metabolic engineering open promising horizons, as the application of tools such as gene editing and omics analyses (transcriptomics and metabolomics) enables the mapping of metabolic pathways and the identification of key targets to optimize biomass production. This facilitates the development of more efficient *H. pluvialis* strains, capable of accumulating higher amounts of astaxanthin and other bioactive compounds. These advances not only enhance productivity but also allow the modulation of nutritional profiles, expanding the possibilities for application in various human food products.

Consequently, the development of new functional foods has gained prominence, and the incorporation of whole or fractionated *H. pluvialis* biomass into a wide range of products—such as meat analogs, fermented beverages, baked goods, and even 3D-printed desserts—has been extensively studied. Beyond the antioxidant and anti-inflammatory activity of astaxanthin, the proteins, polysaccharides, and lipids present in this microalga provide additional benefits, enabling the formulation of foods with functional properties.

Despite the demonstrated potential, several barriers remain to be overcome. The high cost of cultivation, extraction, and purification processes continues to hinder industrial-scale production. In addition, there is a need to better understand the stability of bioactive compounds during processing and to develop solutions that ensure sensory characteristics acceptable to consumers. Regulatory aspects and international standardization also need to be addressed to enable the safe and competitive introduction of products to the market.

## 9. Conclusions

This review highlights that *H. pluvialis* is one of the most promising sources of natural bioactive compounds, primarily due to its capacity to produce astaxanthin, a recognized antioxidant associated with multiple health benefits. In addition to this carotenoid, the microalga provides a comprehensive nutritional profile, including high-value proteins, essential fatty acids, polysaccharides, and vitamins, positioning it as an excellent strategy for the development of functional foods.

While current applications are still mostly concentrated on dietary supplements and natural colorants, there is vast untapped potential for its incorporation into diverse food matrices, including plant-based products, functional beverages, and novel emulsified systems.

Future research should prioritize the optimization of large-scale cultivation and the development of cost-effective extraction technologies that preserve bioactive stability while ensuring environmental sustainability. Industrial perspectives point toward the adoption of omics-based tools, strain engineering, and circular economy approaches as essential strategies to overcome existing bottlenecks. Furthermore, regulatory harmonization and greater consumer awareness will be decisive in accelerating the commercialization of *H. pluvialis*-based ingredients.

Overall, the integration of technological innovation with sustainable production practices will be key to unlocking the full potential of *H. pluvialis* as a multifunctional ingredient, contributing to healthier diets and more resilient food systems.

## Figures and Tables

**Table 1:** Composition of *H. pluvialis* at the green and red stages. Source: Grewe and Griehl (2012) [12]. n.d.: no data.

Composition Content (% DW)	Green Stage	Red Stage
Proteins	29–45	17–25
Lipids (% of total)	20–25	32–37
Neutral lipids	59	51.9–53.5
Phospholipids	23.7	20.6–21.1
Glycolipids	11.5	25.7–26.5
Carbohydrates	15–17	36–40
Carotenoids (% of total)	0.5	2–5
Neoxanthin	8.3	n.d.
Violaxanthin	12.5	n.d.
β-Carotene	16.7	1.0
Lutein	56.3	0.5
Zeaxanthin	6.3	n.d.
Astaxanthin (incl. esters)	n.d.	81.2
Adonixanthin	n.d.	0.4
Adonirubin	n.d.	0.6
Canthaxanthin	n.d.	5.1
Echinenone	n.d.	0.2
Chlorophylls	1.5–2	0

## Data Availability

No datasets were generated or analyzed during the current study.
